# Acquiring a new understanding of illness and agency: a narrative study of recovering from chronic fatigue syndrome

**DOI:** 10.1080/17482631.2023.2223420

**Published:** 2023-06-12

**Authors:** Anne Karen Bakken, Anne Marit Mengshoel, Oddgeir Synnes, Elin Bolle Strand

**Affiliations:** a Centre of Diaconia and Professional Practice, VID Specialized University, Oslo, Norway; b Department for Interdisciplinary Health Sciences, University of Oslo, Oslo, Norway; d Dep of Digital Health Research, Oslo University Hospital, Oslo, Norway

**Keywords:** recovery process, illness narratives, narrative analysis, very severe CFS/ME, qualitative interviews, contested diagnose, personal experiences, persistent physical symptoms

## Abstract

**Background:**

The condition known as chronic fatigue syndrome or myalgic encephalomyelitis (CFS/ME) is poorly understood. Simplified medical models tend to neglect the complexity of illness, contributing to a terrain of uncertainty, dilemmas and predicaments. However, despite pessimistic pictures of no cure and poor prognosis, some patients recover.

**Purpose:**

This study’s purpose is to provide insight into people’s experiences of suffering and recovery from very severe CFS/ME and illuminate understanding of how and why changes became possible.

**Methods:**

Fourteen former patients were interviewed about their experiences of returning to health. A narrative analysis was undertaken to explore participants’ experiences and understandings. We present the result through one participant’s story.

**Results:**

The analysis yielded a common plotline with a distinct turning point. Participants went through a profound narrative shift, change in mindset and subsequent long-time work to actively pursue their own healing. Their narrative understandings of being helpless victims of disease were replaced by a more complex view of causality and illness and a new sense of self-agency developed.

**Discussion:**

We discuss the illness narratives in relation to the disease model and its shortcomings, the different voices dominating the stories at different times in a clinically, conceptually, and emotionally challenging area.

## Introduction

Chronic fatigue syndrome or myalgic encephalomyelitis (CFS/ME) is defined by the presence of a set of symptoms and significant impairments. The diagnosis requires that adequate medical examination shows no evidence of any other condition to sufficiently account for the whole clinical picture (Cortes Rivera et al., 2019; Nacul et al., 2021). Core symptoms are a low threshold of physical and mental fatigability and post exertional malaise accompanied by different complaints including pain, sleep disturbance and cognitive impairment. The affliction has a long history of disputes and changing names and concepts (Lim & Son, 2020). There are disagreements on how to understand, classify and label the illness, and no generally accepted case definition, aetiology, pathophysiology or attainable targets for treatment exist (Wojcik et al., 2011; World Health Organisation, 2022). Full recovery appears to be rare (Bested & Marshall, 2015; Cortes Rivera et al., 2019). One systematic review found the median full recovery rate without systematic intervention to be 5% (Cairns & Hotopf, 2005). Although psychotherapy, mind-body interventions and rehabilitative care has been shown beneficial for some, current guidelines warn against some of these approaches and emphasize that none of them should be considered curative (Gotaas et al., 2021; Khanpour Ardestani et al., 2021; NICE, 2021a; Sharpe et al., 2021). The most severely affected bedbound patients are understudied (Chang et al., 2021; Pendergrast et al., 2016; Sharpe et al., 2021). Current recommendations for the care of patients with very severe CFS/ME aim at minimizing suffering and manage symptoms and their prospects of improvement remain uncertain (Montoya et al., 2021; NICE, 2021a). Nevertheless, personal stories of recovery exist. These stories may embody valuable knowledge on the process of regaining health, which is the topic we set out to explore in this study.

### Narratives of illness and recovery

According to the medical sociologist Arthur Frank, severe illness requires new and more self-conscious solutions to general “body problems”, including bodily predictability and degree of control. Illness involves a loss of the “destination and map” that previously guided life (Frank, 2013).

Severe illness is not only biology out of order but a personal “biographical disruption” (Bury, 1982). The experience of illness calls for understanding and answers to the fundamental questions of illness causation, its likely course and possible alleviation, and for finding a way of relating to it (Kleinman, 1988). Storytelling in illness is a crucial way of making sense of personal experiences, communicating the past and present and predicting and shaping the future (Hydén, 1997). However, finding a new direction through storytelling does not happen independently of available narrative resources (Frank, 2010, 2013). Personal narratives are constructed under the influence of cultural and social contexts, including salient stories of others’ illness experiences, explicit and implicit institutional knowledge and medical models, classification and nosology (Brown et al., 2017; Kirmayer & Sartorius, 2007; Malterud et al., 2015). Moreover, narratives do not only reflect illness experience, but may also contribute to the experience of symptoms and suffering (Frank, 2013; Kleinman, 1988) by providing a model for explanation, attribution and meaning (Kirmayer & Sartorius, 2007). Referring to the medical historian Anne Harrington’s emphasis on mind-body medicine being “a storied world”, Arthur Frank claims that although stories are not the only actors, narratives templates are fundamental resources that “set the terms of thinking, acting and even imagining in this field” (Frank, 2010, pp. 118–119). Mattingly’s notion of “therapeutic emplotment” emphasizes the connection between stories and action, stories’ potential to create experiences and the possible therapeutic value of story-making. Thus, also supported by Riessman (2008), we can think of illness experience as a phenomenon in constant interplay with personal illness narratives and dominating or available narratives at a cultural level. Lack of firm scientific explanations and contested epistemic status of an illness leaves a space for different and possibly conflicting narratives, both within the medical community, in society and at the individual level.

The most available and socially condoned illness narrative in Frank’s typology is what he names the “restitution narrative”, which has the following plotline: “I was healthy, now I’m sick, but I’ll become healthy again”. If not caused by a self-limiting condition, suffering is typically relieved by modern medicine’s promise of finding an explanation and a corresponding cure. Frank describes two other basic types of illness narratives, chaos and quest, recognizing that all three narrative types are present in any single story with varying prominence at different times and contexts and influenced by many factors, including cultural and personal preferences. While the restitution story presupposes that control over disease is necessary to effect recovery, in the chaos narrative the future seems unpredictable and out of all control, the story is falling apart and “the body is imprisoned in the frustrated needs of the moment” (Frank, 2013, p. 98). The quest narrative, on the other hand, portrays the illness experience as a possibility for the person to take agency and attain changes and growth.

### Symptoms in a dominating biomedical worldview

Although it is has long been known that the relationship between conscious experiences of symptoms and indicators of objective physiological dysfunction is highly variable (Beecher, 1956), medical reasoning and practice often do not reflect the complexity of symptom perception. When symptoms persist, and there is no identified underlying disease to cure, contemporary medicine struggles to provide sufficient relief and seems to lack the necessary understanding and tools to help people recover (Cassell, 2004; Crowley-Matoka et al., 2009; Sharpe & Greco, 2019). Despite being common health problems, “Persistent physical symptoms” have no obvious place in diagnostic classifications and tend to be put in residual categories (Jutel, 2010; Rasmussen, 2020). As diagnosis plays a pivotal role in medical epistemology and management, confusion about labels and concepts have clinical and scientific consequences. The collective term “medically unexplained”, traditionally used to describe bodily complaints when the aetiology is unclear, is now widely criticized and in some professional contexts largely abandoned due to conceptual, pragmatic and ethical problems (Creed et al., 2010; Greco, 2012; Jutel, 2010).

Despite advances since George Engel (1977) pointed out the shortfalls of a reductionistic biomedical model and introduced a biopsychosocial view on illness and disease, we still lack clinical language and everyday vocabulary to capture different levels of explanation and multiple causality.

The term “symptom” in a modern medical setting refers to a subjective phenomenon; a percept interpreted to indicate a change of order (Eriksen & Risør, 2014; Malterud et al., 2015). When people bring worry and suffering to a medical encounter, their complaints become “symptoms” and sources of information for a diagnostic process, “sorting out the real from the imagined, the valid from the feigned, the significant from the insignificant, the physical from the psychological” (Jutel & Conrad, 2011, p. 23). Through sorting and classifying in a naturalistic framework, disease can be revealed and managed. However, this approach is often unsuitable for understanding the person’s experience of suffering and insufficient when faced with symptoms with no clear origin (Cassell, 2004; Kirkengen et al., 2016). Patients with suffering that does not fit into the biomedical paradigm may find their illness having an ambiguous social status of validity and legitimacy at the lower end of a tacit prestige hierarchy of medicine (Album & Westin, 2008).

Biomedicine’s inherent mind-body dichotomy and assumptions of linear causality may lead to the simplified inference that symptoms without an objectively verified “organic” explanation must be “psychogenic” (Arnaudo, 2017; Kirkengen et al., 2016; Kirmayer & Gómez-Carrillo, 2019; O’Leary, 2018). If the person localizes the symptom in the body and does not experience any primary mental suffering, this conclusion will likely elicit doubt, uncertainty and feelings of being dismissed, discredited and stigmatized (Lian & Robson, 2017; Nettleton et al., 2005; Salmon, 2007). Furthermore, embedded mind-body dualism reflects a parallel dichotomy: voluntary versus involuntary actions. Explanations tend to dichotomize illness experiences into the ones we can control and the ones we are not responsible for, without accounting for gradations of human agency (Kirmayer & Gómez-Carrillo, 2019). There is a tendency to attribute the locus of agency to the person when the problem is thought to be “psychological” in origin, as opposed to a biological illness mechanism for which the person is not held directly responsible (Kirmayer & Gómez-Carrillo, 2019). Personal agency points to possible wilful solutions to a problem and promises therapeutic potential. On the other hand, this kind of social logic may also tacitly or explicitly lead to moral judgements of responsibility and blame for a problem assumed to be “all in the mind” (Jutel, 2010; Kirmayer & Gómez-Carrillo, 2019). Thus, questions concerning agency, and whether patients can have an impact on the course of their symptoms and illness by controlled actions, remain sensitive and potentially controversial issues. Outcome research shows only a moderate-to-low level of evidence for the efficacy of studied treatments. As is the case in recommendations for CFS/ME, current non-pharmacological therapeutic methods for other persistent symptoms often aim at improvement and coping rather than full recovery (Peter Henningsen, Gündel, et al., 2018; Roenneberg et al., 2019; van Dessel et al., 2014).

### Narrative research on recovery from CFS/ME

Personal experiences of recovery from CFS/ME are rarely studied. Despite solid reasons to supplement evidence-based methodology with a first-person perspective in the study of conditions defined by subjective phenomena (Cassell, 2004; Charon, 2008; Greenhalgh, 1999; Kirkengen et al., 2016; Malterud & Aamland, 2019), former patients’ experiences seem to be an underutilized source of knowledge. Previous work using a narrative approach in the study of CFS/ME has shown the value of co-constructing stories to validate individuals’ experiences of suffering from a contested illness (Bülow, 2004). Whitehead (2006) found a trajectory of dominating narratives starting with “restitution”, followed by “chaos” and then, in recovery, “quest”. Although still having symptoms, people felt they had gained valuable insight through illness and adjusted ways of living and self-identity. In a study of narratives on what it means to recover, Cheshire et al. (2020) display differing understandings, noting that people nuance their definitions of “recovery” to adjust to limited expectations of eliminating all symptoms. Patients seem to doubt that complete recovery exists but believe significant improvement is a viable goal (Devendorf et al., 2018). Brown et al. (2017) found the recovery phase in the experience of CFS/ME to be a move from one problematic status of falling between socially recognized and medically sanctioned categories to another similar status. Studying adolescents, Krabbe et al. (2023) found the experience of recovering to be a demanding process based on a gradually rising body-based self-knowledge.

Hence, stories of full return to health from severely debilitating states of CFS/ME arise as striking negations against the backdrop of poor prognoses and no recommended cure. The main purpose of this paper is to contribute to knowledge on what characterizes stories of radical change from a bedridden state to a healthy life as told by those having recovered from very severe CFS/ME. We aim to provide insight into people’s experiences of suffering and recovery and illuminate understandings of how and why changes became possible.

## Methods

### Participant recruitment and sampling

Sampling was purposeful and criteria based. Criteria were chosen to ensure the inclusion of experiences of distinct change from severe illness to restored health considering previous research showing 1) the conceptual ambiguity of both CFS/ME and “recovery”, 2) the estimates of very low rates of full recovery and 3) the scarce studies showing full recovery from very severe states. Thus, the sample was intentionally homogeneous regarding some contextual factors in which the unit of analysis was experienced. Potential participants were invited via presumed gatekeepers in Norway: a network of healthcare professionals and researchers working in the field, four specialized healthcare institutions and Recovery Norway, an organization of people who have recovered from CFS/ME or illnesses often labelled “medically unexplained”. Via gatekeepers, we requested consent from potential participants to contact them. Twenty-one potential participants registered their interest and were contacted by phone to ensure they were well-informed and assess whether they met the inclusion criteria:

Participants had to report the following:

- previously having suffered from a condition diagnosed as CFS/ME according to the national guidelines at the time and having a medical record that could verify the diagnosis
- having been bedridden and dependent on care over a continuous period of at least three months during the illness, classified as “very severe” (B. M. Carruthers, M. I. van de Sande, K. L. De Meirleir, N. G. Klimas, G. Broderick, T. Mitchell, D. Staines, A. C. P. Powles, et al., 2011; NICE, 2021b)^1^
- having fully recovered from CFS/ME, with “recovered” defined by the participants considering themselves as recovered and participants not reporting symptoms or functions compatible with CFS/ME at the time of the interview

Four men and 10 women (ages 20–78) were included. The median duration of illness was five years (range 2–22). The median time since full recovery was nine years (range 1–12 years). The participants had been completely bedbound, shielded from sensory input and in need of assistance with all basic functions from three months to six years (Table I).

**Table I.** Overview of all participants with background and illness-related information.

Age at interview (decade)	Gender	Social situation at illness onset	Subjective causality of illness	Duration of illness in years (bedridden, in years)
60s	F	In her 40s. Living alone in a rural area, child-free, full-time job	Infection, chronic emotional stress, life events and heavy burden of care	17 (2)
50s	M	In his 30s. Living alone in a city, child-free, higher education, full-time job, high achiever	Chronic disease, acute infections, emotional stress, and heavy workload	6 (4)
60s	F	In her 40s. Divorced, living with children in a city, higher education, full-time job	Physical and mental interaction of causes, not further specified	9 (2)
50s	F	In her 40s. Divorced, living with children in a city, partner, higher education, full-time job	Mild infection (common cold), emotional stress, a heavy burden of care and tension in close relationship	2,1 (2)
30s	M	In his 20s. Married, two children, living in a city, ambitious full-time student	Severe infection, perfectionism and anxiety related to being a high achiever	4 (1,5)
30s	F	<20 Living with parents and siblings in a town, school	Physical and mental interaction of causes, not further specified	3 (2)
40s	F	In her 20s. Living alone in a city, single, no children, student	Performance pressure, physical overload, internal conflicts	10 (3,5)
30s	M	In his 20s. Living alone in a city, single, no children, student	Mononucleosis, heavy workload, performance-oriented, fast living	4 (0,3)
50s	M	In his 30s. Married, children, living in a rural area, full-time job	Accumulated stress, emotional sensitivity, problematic relationships, stressful life events	10 (1,2)
20s	F	<20. Living with parents and siblings in a town, school	Mononucleosis, no known other cause	7 (3)
30s	F	<20. Living with parents and siblings in a rural area, school	Acute viral infection, no known psychological contribution	(1,5)
20s	F	<20. Parents divorced, shared parenting, living in a city, school	Acute viral infection	7 (6)
50s	F	In her 30s. Married, children, living in a city, higher education, full-time job	Acute viral infection	14 (3–4)
70s	F	In her 50s. Married, living in a city, grown-up children, full-time job	Physical injury, protracted convalescence, emotional stress	9 (1,5)

*Note*: This table presents all 14 participants and the heterogeneity of the material as regards age, gender (F=female, M=male), background information at illness onset, duration and assumed causes of illness. By “subjective causality” we mean the participants’ understanding of predisposing and/or triggering causes of their own illness as interpreted through their narratives. The total duration of illness is shown in full years, although onset was gradual for most. Most participants gave a precise time indication of the period they were bedridden, sensory deprived and in need of full-time care, which is given in brackets. For confidentiality reasons, we have omitted precise information.

### Narrative interviews

All participants were interviewed individually and with a narrative approach. The initial plan of doing interviews in face-to-face meetings was changed due to the infection control regulations imposed as a result of the Covid−19 pandemic. The first author conducted all interviews via a digital video communication platform, which met the standards of data protection regulations. Written instructions were presented in advance and the interviewer had ensured that participants had access to the necessary technical equipment and were comfortable with digital encounters.

The interviewer and all participants were familiar with video communication, and all had easy access to a computer, camera and necessary software and the technical skills to carry out the interviews. A set of standard data was collected from each participant (gender, age, marital status, family size, current occupation or studies, time of diagnosis and name of diagnosing institution or physician) at the beginning of the encounter or through the interview (severity and duration of illness and treatment). Interviews lasted 55–115 minutes.

The interviews had three phases: an introduction, the participant’s storied illness and recovery experiences and a dialogue to follow up on events or topics that had come up through their stories. The introduction was prepared and set up to serve several purposes: to inform, to establish rapport and trust and to give direction to the rest of the interview (Brinkmann, 2015; Mishler, 1991). A short presentation of the rationale for conducting the study, the interviewer’s professional background, personal interest and clinical experience in the field, were described as follows:

I have been working as a medical doctor in a multidisciplinary team with patients with CFS/ME for many years. Although we often thought we understood at least some of their struggle and complex illness, we too often were incapable of helping or treating them. Stories of recovery caught my interest and curiosity. I wonder how you experience and understand your return to health. I think these stories can give valuable insight and add important knowledge.

The participants were then asked to tell their stories of recovery in their preferred ways and to include what they regarded as relevant with an optional guide from the interviewer: “One place to start could be at the most severe stage of your suffering.”, generally avoiding interruptions.

Guided by principles for narrative research and clinical medicine, the first author aimed to establish a climate that allowed for storytelling (Charon, 2008; Riessman, 2008). Questions were framed open-ended or in other suitable ways, inviting extended accounts. The interviewer aimed to let the interviewee control the direction, content and pace, using her experience as a clinician and knowledge of the complex illness they had suffered to listen actively without interrupting the flow of their storytelling. Some clarifying and probing questions were asked, led by the individual stories and adjusted to suit the preference and style of the interviewee. The interviews were audio-recorded and transcribed verbatim.

### Narrative analysis

On the basis of narratives’ significance in the experience of illness and recovery (Frank, 2013) and to encompass the chronological arc of experience and meaning, “keeping the story intact” (Riessman, 2008), we decided to use narrative analysis in the interpretation of the data. The four authors have diverging professional backgrounds and different experiences in the fields of narrative research and clinical work and research on CFS/ME and persistent symptoms, constituting an interdisciplinary team for the analysis. All authors read the transcribed interviews, first for an overall familiarization with the data, then to analyse each interview separately. Through dialogical reflection in multiple meetings with the research team, the analysis was broadened, enriched and nuanced.

Drawing on Arthur Frank’s dialogical narrative analysis (Frank, 2012) and the initial reading of interviews, we found that changes in relations between the body, self and society could be explored and illuminated by posing two main analytical questions. We asked how the storytellers position themselves as active agents or “hold their own” through severe illness and recovery. As stories are multivocal and display tensions between larger cultural plots and different versions of the self as an active agent, we also ask how merging or contesting voices are expressed and what different voices can be heard in any single speaker’s voice.

We identified plotlines, narrative understandings, transitions, turning points and how the narratives were formed by available narrative resources and different dominating voices throughout the trajectory of illness and recovery. Common patterns in the stories were recognized. Furthermore, we explored what functions the narratives might serve for the storyteller.

We have chosen to present the results in this paper through one participant’s story. The story’s value as a format of sharing knowledge is well substantiated and serve unsurpassed roles in medical communication (Charon, 2021; Radley & Chamberlain, 2001; Vandenbroucke, 2001). One comprehensive and coherent story allows for wholeness in the presented narrative construction of meaning. We chose Erik’s story because it captures a common pattern of plotline, narrative understandings, transitions, turning point and dominating voices found in all stories in an information rich, nuanced and vivid way. Several non-shared aspects and experiences, e.g., perceived causes, onset and duration of illness, duration of recovery and degrees of setbacks, were regarded as less significant for the aim of this paper. Our telling is a result of careful listening and co-constructing of a narrative through a dialogical reflexive and iterative process, with the intent to keep and convey the story’s vividness, authenticity and to “let the story breath” (Frank, 2010).

### Trustworthiness

Narratives do not establish “the truth” of events or experiences, but rather what a person finds relevant for providing meaning of experiences (Denzin & Lincoln, 2000; Riessman, 2008). To strengthen confidence in participants’ telling and establish a plausible interpretation of stories, we have aimed at transparency by making explicit methodological decisions and describing how interpretations were shaped through a thorough and iterative reflexive process in a multidisciplinary research team. Stories are interpreted with attention to language and context, comparison within data and in dialogue with theory to arrive at a balanced and nuanced understanding (Riessman, 2008). Member checking was done to ensure ethical data protection, but also to build trustworthiness. As Riessman (2008) points out, discussions about trustworthiness can easily descend into heated debates on generalizability. A compelling story’s persuasive potential may evoke eligible opposition. We acknowledge context-dependent limitation of transferability but also the pivotal role case based narrative research may play (Flyvbjerg, 2004; Nissen & Wynn, 2014).

## Ethics

The Regional Committee for Medical Research Ethics, and the Norwegian Centre for Research Data approved the study (REK: 192119, NSD: 342533). Data used in this paper were cleaned to remove personal identifiers. The story used in this paper is further modified to remove contextual identifiers. Since participants had faced unusual life events within a limited population, some possible identifiers may remain. The participant who originally shared the story used has read it in its final version, acknowledged its authenticity and consented to publication. Issues of anonymity and confidentiality were explicitly discussed with all participants, and we have followed ethical standards concerning voluntary participation, informed consent, right to withdraw, respect for participants and their contribution and minimizing potential for harm.

When conducting interviews through a digital platform turned out to be the only option, certain ethical issues arose as recently described by Maldonado-Castellanos and Barrios (2023) concerning privacy, confidentiality, accuracy of information and technological literacy. The participants received thorough information ensuring that only the interviewer could hear and watch the screen, and that the digital platform was secure, and approved for research.

## Patient involvement

Former patients, who had lived experiences of both illness and recovery from CFS/ME, were involved with the aim of strengthening both the ethical standard and the value of scientific knowledge development. Recovery Norway (RN), an organization of people who have recovered from CFS/ME and similar health issues, acted as a formal collaborator. RN contributed with inputs in the very first phase of idea development and planning of the study, although they did not collaborate in designing the study or in development of interview guides. RN was one of several sources for the recruitment of participants. Data analysis was carried out without the involvement of RN representatives, patients or former patients.

## Results

All participants told their stories coherently and continuously, looking back through the lenses of regained health and sense of self-agency. The analysis revealed a common plotline found in all the stories. Despite differences in perceived causes, onset and duration, all the stories shared a trajectory in which the main sequence of events from the development of illness and a long period of severe, debilitating suffering was followed by a distinct turning point towards recovery. The turning point involved a profound shift in interpretation and perspective in their illness narratives, as in Erik’s story:

I have had several stories about my own illness, different ways to understand it … At one point it changed radically and that was what I needed to get out of it and get well … I think deep down what happened was that I replaced my old worldview. It took great effort.

Informants viewed this demanding transformative narrative change to be necessary, although not sufficient, to enable recovery. We use Erik’s story to illustrate the typical development of the illness narratives in our material through four phases: 1) developing illness: in search of an explanation, 2) severe illness: deteriorated and bedridden, 3) turning point: cracking worldview and new discovery 4) healing: holding on to new worldview. After five years of illness, Erik had been in good health for ten years at the time of the interview.

### In search of an explanation for persisting symptoms—a long-haul pursuit leading down a blind alley

Erik recalled regarding himself as a strong, hardworking, and successful student when he suddenly fell ill with mononucleosis and two subsequent serious infections, requiring hospitalizations. Despite treatment of infections and a good prognosis, he gradually deteriorated, and fatigue, pain, flu-like symptoms, and exertional intolerance narrowed his life more and more.

I was just slowly getting sicker and sicker. I felt disconnected from my senses, dizzy, and the exhaustion grew heavier and heavier, and I was constantly being punished [by my body] for what I was doing.

The change in life was a shock to him. He lost a sense of control and self-confidence when his life was in the hands of medical experts. In an attempt to make sense of his experiences, he assumed there was some underlying, undetected, dangerous disease. Long-term comprehensive medical examinations in search for disease reinforced his suspicion, and lack of explanations was confusing. Uncertainty added extra burden to suffering. Illness taught him to listen more carefully to his body, although not necessarily as a conscious and deliberate act. On later reflection, Erik assumed that his increased awareness of bodily signals had started as a way of regaining control when anticipation of lasting illness was inevitable.

All that youthful feeling of immortality was just blown away … . Because I had experienced several times over the course of a few months that when I thought I was on the way to recovery, I suddenly got really sick again. So, I was waiting for it, I was just waiting to get sick again. And I think it became a habit stuck very deep inside me, to listen carefully to my body to detect if I was about to get sick again: Do I have a fever? Do I have pain there? Am I tired?

It is only when I look back that I can see what was probably going on inside me, but I was not aware of it myself at the time … . I was the model student who was struck by an illness. Boom! An unpreventable, devastating physical disease.

Eventually, the medical conclusion was that no disease could be objectively verified, mental illness had been ruled out and the diagnosis was CFS/ME. The conclusion was a relief, offering reassurance, validation to the experience of suffering from a “real” physical condition and giving medical legitimacy for sickness behaviour. But in hindsight, Erik claimed the diagnosis was a double-edged sword. The terms “chronic” and “encephalomyelitis” signalled infinity and indicated irreversible physical damage. And since the label CFS/ME did not imply a univocal explanation, effective treatment or prediction of the future, insecurity persisted. Some explanations made more sense than others but gave little hope.

One doctor … I think he meant well and deliberately tried to downplay [the diagnosis]. But I didn’t get any explanation, and I was so desperate for an explanation. I wanted to know what was wrong with me. And when [another doctor] said “this is probably a physical, neurological disease, maybe there’s something wrong with the mitochondria in your cells”, then everything fell into place. It made perfect sense; it fitted my feeling”

… but the doctors gave me little hope of recovering from ME. One consultant said, “We’re sorry, there is nothing we can do to help you”.

He had heard the same story from health personnel, official patient education, patient support groups and mass media: CFS/ME is a serious “multisystemic” disease with an unknown cause, poorly understood biological mechanisms, no evidence-based treatment available and a poor or uncertain prognosis. Unclear information from doctors and the uncertainty that permeated both scientific and lay literature about the illness made life unpredictable. The general instruction was not to overdo things, although the rationale behind this vague advice and the aim of “saving energy” remained elusive. On reflection, Erik said the advice had been helpful in the moment, but useless in the long run.

I was very active seeking information online. And I tended to grab onto the darkest, the gloomiest scenarios that fitted the way I felt … I found numbers and was horrified! No more than two to four out of a hundred ever recovered completely, and many got worse and were bedridden forever! The picture of the future was all dark …

The information I received from healthcare … and what I found on websites and online patient groups, it was all about adjusting activities, so I did. It’s no treatment, but it’s a measure. I was advised to listen to my body and making sure not to overload … . One doctor helped me set up a plan to ensure I didn’t spend more than 70% of my energy … I had to find out what I could tolerate and what made me worse. So, I was very careful and conscious. While I was continuously deteriorating … Later, I’ve thought a lot about “pacing”. I got the advice I was looking for, that fitted my experience. I know it helps some. But I paced myself to bed!

Signals from the body had been the most substantial information to lean on. Symptoms became the main compass to prevent worsening and limit harm. The only way he knew to alleviate symptoms was to restrict activity and conserve energy. All his bodily experiences confirmed his convictions.

The disease was inflicted upon me, beyond my control. I never doubted, it *was* the truth, it was my reality. It became such a fine-grained, detailed web of truths that was a hard-wired part of my life for many years…I *knew* I didn’t tolerate any movement! If I made a move, pushed my limits … . I crashed … the proofs of my reality were so physical! Most of the imperative to avoid activity came from myself.

A referral to mental health service was in Erik’s eyes at best an annoying required procedure to be entitled to welfare benefits. Psychiatrists and psychologists had defined the condition as outside their domain, declared their lack of knowledge and confirmed that psychological interventions could not cure but possibly help him cope with a physical disease like CFS/ME. When conventional healthcare failed to provide an explanation and solution, Erik and his family started their own ceaseless hunt for causes and remedy. Although the act of “doing something” helped sustain hope and meaning, no medical or alternative treatments gave him any relief from his symptoms.

I tried everything we could find, including papaya extract, magnetic soles, healer, you name it … Many shots in the dark … A nutrition expert had that “leaky gut theory” and I was put on a diet … I placed my trust in all this, but it didn’t work.

### Deteriorated and bedridden—enduring agony, relating to reality, and rejecting non-sense

Over months and years, symptoms and impairment persisted, and Erik became increasingly constrained, physically, mentally and socially. When he was most severely affected, Erik was completely debilitated and bedridden. Even minor stimuli of any kind led to sensory overload. He had laid shielded in utter darkness and silence wearing a blindfold and soundproof earmuffs. Windows and walls were reinforced to shut out all light and noise. He was unable to eat solid food and needed assistance with all basic functions. Any movement or handling triggered pain and exhaustion, so nutrition and personal hygiene were poor. Communication was limited to a minimum as he could only whisper and allowed only the strictly necessary visits of caregivers. This state of total confinement to bed persisted for 15 months.

I lay there…staring out in the dark … and even then … I had pain everywhere, and I could not bear to be touched. … it was like a constant hurricane inside.

… every time I was exposed to a little strain, I got even worse. Maybe for many days … If I had tried to lift my head a little in my sleep, I crashed for two or three days … . At least, that was the connection I saw at the time.

When the caregiver burden exceeded what family members were able to handle, Erik was moved to a nursing home. Deprived of close relationships and dignity, he still had hope for survival, but he felt he was near the edge of existence and had “nothing left to lose”.

Erik’s relatives collected personal testimonies from former patients who claimed they had recovered after attending a mental training programme. These stories had triggered curiosity, but also confusion and resentment since the stories had been incomprehensible and incompatible with Erik’s own conviction. He remembered rejecting the rationale behind the mental training programme as nonsense, unscientific and far-fetched. Strong immediate resistance and fear of being misled to ignore symptoms and getting worse were Erik’s overriding feelings. Although he had not denied the general idea that mental processes affect the body, this mechanism did not feel relevant in his case, and such suggestions were provoking.

According to a research article I had read before I got really sick, ME sufferers who were open to the fact that psychological factors could play a role had a greater chance of recovering … . And I thought, “Sure! Among those who believe that psychological factors play a role, there will certainly be people who are just mentally ill. So, they don’t have ME. They are not sick like me”. It made me angry. I needed to be taken seriously!

In Erik’s worldview, relating to the stories of recovery would have implied accepting that his symptoms were imaginary, “unreal” or “all in the mind”, which was inconceivable since his body “told him otherwise”. He had seen no need for psychological adjustments or help to cope and recalled his defence with self-deprecation:

I just related to reality, I coped with my condition damn well. I saw myself as mentally very strong, enduring that agony. I was not afraid, but I knew that if I did this and that, I got worse, so I didn’t do it.

### Cracking worldview—navigating unknown, rugged terrain to new discovery

Despite reluctance, the feeling of having nothing left to lose made Erik accept an individually adjusted version of the mental training programme. For several hours on three consecutive days, a coach had come to his room and presented a model of mind-brain-body connections and possible ways to actively modify symptoms by self-coaching techniques. The coach validated his symptoms as real, and at the same time introduced to the possibility that what he experienced as symptoms could be a false alarm rather than necessarily signs of disease. He learned that by actively changing his interpretations of symptoms every time they appeared, direct attention and reduce distress, he could influence the “production” of symptoms. The techniques included certain patterns of physical movements that accentuated self-instructions aiming to empower him to take control and “change his mind”. In this logic, such change of conscious experience would inevitably also involve changes at the biological level, including neurological and hormonal function.

At first, he attended the lessons with scepticism, resistance, fear and doubt. His persuasion was so deeply rooted that any alternative reality seemed pervasively counterintuitive and threatening. The initial change was accompanied by a fierce inner struggle.

It was absolutely ridiculous … extremely hard to imagine that someone would come into my room and sit and talk to me for three days; it seemed impossible … I was positively convinced that if this doesn’t work and it doesn’t work quickly, then I will end up in a coma or … die … .

… much of the things the coach said made sense, but … at the start of day two, I was completely locked in my old world again, I felt terrible, I was nauseous and weak and could hardly speak … I had to tell them to dim the light again … and I asked the coach to speak softer.

However, change gradually became more of a bodily discovery than a theoretical concept. Along with altered thinking and movement, experiences also changed. Erik understood this as a two-way interaction: feedback from his body was the necessary tangible evidence he needed to build hope and trust in his new beliefs, and this newly acquired confidence made movement possible. For every step of starting to tolerate light, sound, eating, sitting and putting his feet on the floor, he was pushed to challenge himself while practising techniques. In just a few hours he had made considerable functional progress.

I started to realise that I could influence my own state of mind, which in turn meant affecting the intensity of pain and fatigue

… and when I took that leap, from beginning to believe, to actually dare to *know* that it was safe to raise the head end of the bed and take down the extra light blocking curtains. And it was safe to start talking in a normal voice again … . The weird thing was, when I *knew* it was that way, it somehow became true.

In Erik’s opinion, the great momentum of the initial change in mindset and illness beliefs and a time-compressed training programme were essential steps towards recovery. With secure support from a person with contagious determination and firm conviction, he perceived enough proof of a new reality without being overwhelmed by doubt. Looking back, he thought the critical moment could easily have slipped away.

… the fact that I was challenged so completely was crucial … . I had to be so brutally thrown into it … If I was to do changes step-by-step over a longer period, I would have had too much time to think … I just had to throw it all away … . And, at the end of the second day, when I managed to let go of the worldview that had occupied my mind for so many years … then the rest was straightforward in comparison.

Over the course of hours and days, Erik’s former understanding of being a passive victim of disease was replaced by trust in the possibility of taking on the role of an active agent with the ability to modify symptoms and take back some control of his body. While he actively explored new interpretations of his own experiences, the experience itself started to change. The transition was imprinted in Erik’s memory as a major leap and irreversible turn in his life story.

### The long and tortuous path to health—holding on to the new worldview

The main turning point in Erik’s story involved an intense emotional experience of relief and power. The following physical rehabilitation and consolidation of a new mindset, however, took time and required effort and endurance. His grip on the new perspective of reality was fragile. He remembered episodes in which he felt he was close to the tipping point again, but with help from specific techniques, he managed to stick to his new conviction. These techniques had to be repeated “many hundred times a day” for weeks before it gradually became automatized. After some months, he considered himself fully recovered, taking up activities and a life with his family again.

It is often presented as too good to be true that you take a three-day course, and it will change everything. … It wasn’t like I took the three-day course and things were fine. I had to practice the exercises constantly …

I struggled; I had a hard time. It was very difficult to hold onto my new worldview, and I began to wonder “have I pushed myself too much, will I get sick again?”, and that thought in itself was very, very destructive, and it could easily escalate. But … I managed to tilt myself down on the right side.

Now it happens very rarely. But still, there are times when I wonder if I have symptoms … .it is interesting that there is still something underneath that can take me back there and remember a little too much… I have to make an effort to get out of it again …

For years afterwards, he tried to avoid conversations about his former illness and contact with patients with CFS/ME, patient organizations and patient advocacy groups because he was afraid his old understanding would be reactivated, triggering symptoms and relapse.

At the time of the interview, an ongoing, heated public debate about CFS/ME engaged Erik. He had heard stories from patients and experts who reported the harmful effects of mentally based interventions for CFS/ME. If these stories had been available to him when he was ill, he doubted he would have dared to make a move and possibly would still be bedridden. Warnings of danger and damage would have fitted perfectly with his experience and understanding at the time. Erik tried to make sense of this apparent contradiction of totally opposite experiences elicited by the same kind of intervention. He emphasized that he did not claim to have “the whole truth” about CFS/ME or the solution for everyone. On reflection, he noted that the process he had been through was a “high-risk transformation journey”. To be able to proceed all the way past the tipping point of fundamental change, Erik had needed both the knowledge of an alternative constructive mental model of his illness and all preconditions to be able to integrate and benefit from it. What these preconditions were in his case was still an open question to him.

You can’t just be deprived of your whole worldview without replacing it with a more helpful alternative—that is dangerous, of course you will get worse.

Erik was confident that he had the power to prevent a relapse. His new understanding did not imply that the illness was purely psychological in nature, “made up” or unreal, but that it was less predetermined by unpredictable and uncontrollable physiological processes.

## Discussion

Our point of departure for the following discussion is the disease model and its limitations in approach to persistent symptoms. Next, we discuss participants’ narrative understandings, how one narrative understanding of illness, self, and body was replaced by a different one, and what voices are prominent through the trajectory of illness and recovery. Finally, we briefly discuss interpretations of recovery stories and changes in the context of a contested field.

### A disease model—promises and predicaments

When severe pain and fatigue brought the participants to seek medical care, they sought to understand why they were ill and expected medical evaluation to reveal the cause. This is in line with assumptions of modern medicine and premises for practice of a “disease model” commonly run by medical experts to determine the causes of suffering and provide a remedy (Johansen & Risor, 2017; Jutel, 2010; Kirkengen et al., 2016): 1) The reality of the first-person perspective (illness) is distinct from the reality of observable bodily pathology (disease). 2) Subjective experiences, regarded in the medical context as “symptoms”, are thought to be generated by bottom-up processes, and illness is understood to be an *effect* of disease. 3) Symptoms (e.g., pain and fatigue) guide the search for explanations primarily in the physical realm. The expected outcome of this process is typically an objectively verified diagnosis, which allows for a causal explanation of symptoms, a prognosis and preferably a cure. Seeking a medical evaluation of suffering typically involves accepting the disease model with its logic and terms and medicine’s priority of explaining illness by detectable disease. With this narrative, modern medicine has had success for centuries. Despite challenges and criticism, the voice of a naturalistic framework is still strong and reflected in diagnostic classification, healthcare structure, medical practice and public expectations (Kirkengen et al., 2016).

However, limits of the disease model are clearly demonstrated through the participants’ stories. When they suffered severely despite tests indicating that their bodies were healthy, difficulties and dilemmas arose. First, the disease model’s assumptions may promote endless and repeated medical investigations, driven by medicine’s obligation to find a resolution, fear of missing a diagnosis, the patient’s demand for an explanation or the physician’s belief that negative test results will provide reassurance and relief for the patient. This process is demanding, expensive and often non-conclusive (Hatcher & Arroll, 2008; Kube et al., 2020). For Erik, the medical examinations were both energy draining and fear provoking as he inferred that a never-ending investigation was part and parcel of suspected severe disease. Furthermore, as claimed by Arthur Frank, modern professionalism still requires the patient’s narrative surrender to medicine, meaning that patients’ stories come to be largely dependent on medical terms and explanations (Frank, 2013). If medical expertise is lost for words, so is the patient. When participants’ hope for a medical restitution narrative was ebbing, the medical voice resigned or became unclear, and understanding of his illness remained elusive. Without necessarily meaning to dismiss the patient’s subjective reality, the physician may convey a presumably positive message of no (severe) disease. But the disease model does not help to comprehend an illness without an evident cause, and Erik was left with unsettled questions of “why me?” and “what can be done?”, crucial issues when illness strikes (Kleinman, 1988). He was “desperate” to be understood, but just as much to understand. Moreover, we can hear troublesome countering voices lurking beneath the surfaces of stories, questioning the moral status of the illness, asking where to place responsibility. Most of the participants, especially before receiving the diagnosis, felt they had to protect themselves from various experiences of social sanctions, adding to the burden of illness, insecurity and loss of control. Inferred from the mind-body dichotomy and failure to identify disease pathology that corresponds to illness, their experiences could be dismissed as “unreal”, “all in the mind” or “purely psychological”. People with different kinds of persistent symptoms seem to be particularly exposed to disbelief, humiliation, stigmatization and marginalization (Kirmayer et al., 2004; Ko et al., 2022; Lian & Robson, 2017; Lipsitt et al., 2015; Nettleton et al., 2005).

### A restitution narrative but no hope for a cure in sight

The diagnosis of CFS/ME represented an ostensible antidote to threatening chaos, a legitimate disease and potentially a new restitution narrative for the participants to hold onto. When Erik was told that there was “something wrong with his mitochondria”, he was relieved. A specific explanation that made sense was better than uncertainty. A biomedical term validated experiences and countered allusions to psychological illness, guilt and implicit or explicit accusations of malingering or exaggeration. In addition to the function of guiding and defining medical care and professionalism, a diagnosis “provides structure to a narrative of dysfunction … and imposes official order” (Jutel, 2009, pp. 278–279).

The participants shared a narrative understanding of being victims of a severe and mysterious physical disease with an uncertain prognosis and no known cause or cure. They envisioned a destructive biological process unfolding in the body, hidden and inaccessible to the affected person. Their stories show an understanding of uncontrollable, lasting and debilitating disease confirmed by solid physical symptoms and doctors’ explanations of biological mechanisms. Voices of medical experts, scientific literature, patient organizations, news media and online patient fora dominated. Participants had no notion or experiences of themselves being able to influence dysfunctional biological processes or modify their perception of symptoms to improve. Erik held his own by listening to his body, following the symptoms’ demands as an intuitive behavioural response. The interwoven forces of severe symptoms and the illness narrative imposed on ill persons allowed for minimal degrees of freedom. Participants felt stuck in a defensive response of predicting and preventing symptoms to avoid deterioration and maintain a minimal sense of control. Symptoms and perceived energy depletion impelled them to reduce activity and preserve energy. Although possibly adaptive behaviour in the beginning, at some point Erik believes he “paced [himself] to bed”. He was caught up in his own story, rendering other stories incomprehensible or beyond reach (Frank, 2010).

The label CFS/ME may implicitly have promised a narrative understanding resembling that of a well-defined disease within the biomedical paradigm. But since the diagnosis provided neither a sufficient explanation, an effective treatment nor a prediction of the future, participants’ fear and hopelessness persisted. At any rate, the diagnosis does not seem to fully protect patients from social sanctions of the medically unexplained (Cheshire et al., 2020; Fennell et al., 2021). On the contrary, there is substantial evidence that many patients with CFS/ME are exposed to negative attitudes, do not feel understood and respected in healthcare encounters as well as more broadly in society and may be particularly susceptible to the phenomena described in the literature as “epistemic injustice” (Blease et al., 2017; Froehlich et al., 2021). Sharpe and Greco (2019) address “illness without verified disease” as a paradox in the context of CFS/ME. They argue that society’s tendency to distrust or devalue patients’ experiences may result in one of two possible routes when attempting to escape this paradox. Since both propositions “this is a real illness” and “this is not a disease” cannot be simultaneously true and valid, given the disease model, there will be those who deny the reality of the illness on the one hand and those who believe the illness is a result of a disease that medical science has not yet discovered on the other. To sustain a restitution narrative, participants in this study had to put their trust in future scientific breakthroughs for a cause to be discovered and a cure to be invented, surrendering to the biomedical narrative.

### Change and a new narrative understanding

Participants’ description of the initiation of change towards recovery, as intense, emotional and momentous experiences, resemble what Mattingly and Lawlor (2001) denote as “healing dramas” with transformative potential. These eventful moments are narrated in detail by participants, burned into the memory as happening when something vital is at stake and accompanied by a strong emotional experience.

The transition between two diverging narrative understandings and a corresponding change in mindset involved participants’ access to new resources, which broadened their interpretative framework and enhanced their capacity to hold their own. In Erik’s words, a total shift in “worldview” was necessary to enable recovery. A mind-body intervention had introduced a compelling counter-voice that soon became salient, trustworthy and decisive, despite initial resistance. The newformed narrative provided a complex and nuanced view of illness and causality, released more degrees of freedom and allowed for new bodily experiences. Two main principles made the new narrative a valuable map for participants to navigate out of illness. The first tenet was that symptoms never accurately reflect the nature of the underlying pathophysiology. Secondly, although a significant part of the information processing involved in perception occurs beyond conscious awareness, it is possible for a person to intentionally influence the shaping of symptoms. The new map showed a way to new interpretation of sensations, options for self-regulation and strengthened sense of self-agency.

Explorative journeys and hard determined work towards health constituted the heart of participants’ stories from this point on. Voices in search of the possible origin of disease weakened, and the quest to identify any symptom as an opportunity to initiate change dominated. On the surface it might seem that our study contradicts a gradual and non-linear process as highlighted by Krabbe et al. (2023). However, we would argue that our findings rather expand the understanding by recognition of distinct and significant turning points, suggesting that such watersheds could possibly be of particular importance in some recovery processes.

A clinically important question is what it may require, if possible, to stop the illness development from going so far and start recovering in an earlier stage. There are suggestions in our material that two apparently opposite approaches by health care professionals, i.e., pushing self-agency too hard or too softly may be experienced as perpetuating factors for illness. We support further exploration on this issue in future studies, e.g., asking what it takes to achieve an acceptable balance between validation of suffering, support and challenge and if such a balance may promote an experience of self-agency.

#### Proposed models of perception and persistence of symptom

The experiences of an acquired sense of control and receding symptoms is in line with a recently developed comprehensive framework of multiple causality, integrating knowledge derived from previous empirical research and a relatively new neuroscientific theory to explain how symptoms come about and may persist (Henningsen, Gündel, et al., 2018; Kube et al., 2020; Van den Bergh et al., 2017). The predictive processing theory is proposed to contribute to the understanding of interoception, i.e., the process of sensing and perceiving the inner state of the body. The theory suggests that conscious phenomena of symptoms do not primarily emerge by bottom-up processes but as a result of continuous interplay between the brain’s predictions about the state of the body, the input through the sensory apparatus and multiple modulating factors (e.g., attention, affective state, perceived threat and previous experiences) with impact at different levels in the process. The highly variable correlations between neuronal input from peripheral sense organs and conscious percepts shown in experimental studies, might possibly be explained by this model (Van den Bergh et al., 2017). Furthermore, the theory applies whether a person has a well-defined disease or not, thus blurring the line between “normal” and “pathological” interoception and possibly reducing the stigma.

Moreover, since the process leading to consciously experienced symptoms happens outside awareness and is not available for introspection, suggested interventions aim at a more helpful differentiation of sensations, breaking the “sensing is believing” cycle and encouraging installation of new neural models by targeted and repeated learning (Van den Bergh et al., 2017). The participants in this study experienced the “mind-body-training” as effective and motivating, without feeling blamed as the production of symptoms and new resources had been inaccessible to them until the turning point.

### Public voices and counter-narratives of recovery stories

Telling stories about full recovery from severe illness do not come without a cost. Participants experienced opposition, discrediting of their stories and the feeling of being caught in the crossfire of heated public and scientific debates about CFS/ME. Erik described the strong warnings against mind-body-based interventions as confusing and potentially disruptive to recovery. He was forced to contend with the fragility of his new worldview, vulnerable to disturbance by compelling counter-voices. Participants felt the need to protect themselves from public judgements and the influence of countering voices, not only to prevent setbacks but also to avoid some responses they experienced as accusatory and hostile. Participants were exposed to attitudes they interpreted as disbelief and suspicion, resembling their previous experiences of epistemic harm while being ill.

Former patients’ experiences of their stories being doubted and dismissed in public could reveal critical issues related to controversies in the field. The reported adverse reactions concerning their former or current state of health were mainly of three kinds: doubt if they had been diagnosed correctly or if they had “real” CFS/ME, doubt if they had really recovered and doubt regarding the way they understood and explained their recovery. Additionally, some felt accused of fabricating stories to promote specific interventions.

Despite the diagnosis being defined by specific symptoms, i.e., by phenomena described from a first-person perspective, the ontological status of the medical entity of CFS/ME remains contested (Iacobucci, 2021; Karfakis, 2018; Sharpe & Greco, 2019). Following the logic of the disease model, a pathophysiological origin of the specific set of symptoms named CFS/ME is presumed to exist, although it is not yet charted. If this hypothesized underlying biological process is supposed to be unaffected by mind-body-based interventions, the recovery stories appear paradoxical. Arguments against the credibility of former patients’ narratives could be interpreted as a way to resolve the paradox by claiming that “real” CFS/ME should be reserved to denote a disease inaccessible to influence by mind or behaviour. Thus, if “real” CFS/ME caused their illness, patients had either not recovered, or recovery could be explained by a natural trajectory erroneously attributed to cognitive and behavioural change. This reasoning can find support in literature warning against mind-body interventions (Twisk & Maes, 2009) and give rise to counter-narratives about patients who, through mind-body interventions, are either misled into believing they have recovered, are manipulated to say they have no symptoms despite suffering or have been misdiagnosed in the first place.

Assuming the authenticity of the stories, independently of how to conceive the diagnosis, a suggested hypothesis is that the persisting physical symptoms and suffering were diminished and disappeared as a result of 1) the participants’ access to a new narrative understanding, which elicited a new mindset and released self-agency and positive expectations in combination with 2) intensive and long-time use of specific mind-body techniques with an impact on the interoceptive process of generating symptoms and 3) a long-time rehabilitation process to consolidate learning and restore health.

In his book *Letting Stories Breathe*, Arthur Frank writes,

Who uses a story to hold their own, and how the story does that, are crucial questions. But it must always be complemented by the question of whom the story renders vulnerable: Who now has an increased problem of holding their own, once the story has been told?

(Frank, 2010)

We acknowledge that former patients’ stories may not be useful for all patients with severe, persistent symptoms and diagnosed CFS/ME to hold their own. Since different narrative understandings of CFS/ME are seemingly incommensurable and intersect at a troubled frontier, the story told in this paper may even leave some people more vulnerable. Erik’s story inevitably raises the question of what is potentially under the individual’s conscious control. While the story may facilitate opportunities for some, it may make others feel violated. Although it is hardly controversial that a complex interaction of factors at the biological, personal and social levels may limit the degree of agency, there is a human tendency to default binary thinking : wilful or not (Kirmayer & Gómez-Carrillo, 2019). We emphasize that the recovery processes we have described were never experienced as a simple matter of will and determination.

## Conclusion

This narrative study on experiences of recovering from severe and debilitating CFS/ME, indicates that a fundamental change in participants understanding of illness and recovery, together with a long-term gradual learning process, was necessary to create their way out of CFS/ME.

Our analysis shows a narrative of complex causality and illness as an interplay of biological, psychological, social and cultural aspects as opposed to a simplistic disease model. Participants’ insight into this complexity appears to have contributed to their sense of self-agency and ability to influence their own experiences of illness. Our results are also possibly in line with recently developed generic models of the persistence of symptoms, including proposed theory on interoception. Controversies in the scientific field and a polarized public debate add burden to afflicted persons not only during illness but also in recovery.

A natural progression of this work is to analyse the moments of change in more detail, exploring various elements plausibly facilitating and moderating the relief from symptoms and the regaining of health.

## Note

1. There may be marked fluctuations of symptoms, making severity definitions difficult. Classification is described similarly by B. M. Carruthers, van de Sande, De Meirleir, Klimas, Broderick, Mitchell, Staines, Powles, et al. (2011). Myalgic encephalomyelitis: International Consensus Criteria. *Journal of Internal Medicine*, *270*(4), 327–338. https://doi.org/10.1111/j.1365–2796.2011.02428.x and NICE. (2021b). *Severity of ME/CFS*. National Institute for Health and Care Excellence NICE Retrieved December 18, 2022 from https://www.nice.org.uk/guidance/ng206/chapter/Recommendations#diagnosis: **Mild (an approximate 50% reduction in pre-illness activity level), moderate (mostly housebound), severe (mostly bedridden) or very severe (totally bedridden and need help with basic functions)**
